# ERGA-BGE reference genome of *Hirudo verbana, *a once neglected freshwater haematophagous European medicinal leech

**DOI:** 10.12688/openreseurope.21672.1

**Published:** 2025-12-22

**Authors:** Alejandro Manzano-Marín, Astrid Böhne, Rita Monteiro, Thomas Marcussen, Torsten H. Struck, Rebekah A. Oomen, Wellcome Sanger Institute Tree of Life Management, Samples and Laboratory Team, Aarushi Vaidya, Abitha Thomas, Adam Bates, Aleksandra Bliznina, Alex Makunin, Amit Vishwakumar, Amy Denton, Andy Griffiths, Anna Kovalevskaia, Arif Maulana, Benjamin Jackson, Cam Muyo, Caroline Howard, Charlotte Wright, Chloe Leech, Chris Laumer, Clare Cornwell, Claudia Weber, David Rowland, Ed Symons, Edel Sheerin, Elizabeth Sinclair, Ellen Cameron, Emma Teeling, Emmelien Vancaester, Erna King, Filipa Sampaio, Gene Myers, Graeme Oatley, Haddijatou Mbye, Halyna Yatsenko, Haoyu Niu, Ian Still, Isabelle Clayton-Lucey, Jack Monaghan, Jamie Davis, Jess Bernard, Jessica Thomas-Thorpe, Jessie Jay, Joana Meier, Jonah Walker, Juan Pablo Narváez Gomez, Kamil S Jaron, Keith Porter, Kerstin Howe, Lauma Ramona, Leah Bacon, Lewis Stevens, Liam Prestwood, Lora Downes, Lucy Kitchin, Luke Lythgoe, Maja Todorovic, Manuel Batista, Manuela Kieninger, Mara Lawniczak, Marcela Uliano-Silva, Maria Morra, Mark Blaxter, Martha Mulongo, Matthew Berriman, Max Brown, Molly Carter, Nancy Holroyd, Nicola Chapman, Paul Flicek, Priyanka Sethuk Raman, Radka Platte, Raquel Juliana Vionette do Amaral, Rebecca O'Brien, Richard Durbin, Robina Heathcote, Sam Ebdon, Sinead Calnan, Sophie Potter, Stephanie Fagan, Theodora Anderson, Victoria McKenna, Witek Morek, Yan Liang, Wellcome Sanger Institute Scientific Operations: Sequencing Operations, Abby Crackett, Abdulrahman Tuameh, Alexander Dove, Alexander Hatton, Alice Linsdell, Ana Monteiro, Barbora Pardubska, Ben Farr, Callum Murray, Carlos Jimenez Verdejo, Caroline Mitchell, Chris Henderson, Craig Corton, Danni Weldon, Elizabeth Easthope, Elliott Trigg, Emily Abraham, Emily Gallagher, Emma Dawson, Emma Memune Taluy, Esther Mellado Gomez, Filipa Sampaio, Francesco Iacoviello, Hannah Hanks, Harriet Johnson, Harriet Ninsiima, Henry Mallalieu, Hermione Blomfield-Smith, Ifeoluwapo Joshua, Iraad Bronner, Irene Fabiola Roman Maldonado, Jacqui Brown, James Du Preez, James Mack, James Uphill, James Watts, John Tushabe, Karen Oliver, Karolina Kujawa, Leanne Morrow, Lesley Shirley, Lucy Kitchin, Maariyah Rashid, Mary-Ann Santosh, Mia Franulovic, Michael A. Quail, Michelle Smith, Naomi R. Park, Neil Marriot, Nicholas Redshaw, Paul Heath, Ritoza Das, Robert Newell, Robin Moll, Sally Linsdell, Sarah Holmes, Scott Thurston, Shelly-Ann Coutts, Sophia Uvarova, Tavis Mason, Timi Adewumi, Tobi Ajenifuja, Tracey-jane Chillingworth, Tristram Bellerby, William Knight, Yousra Belattar, Zoe Goate, Wellcome Sanger Institute Tree of Life Core Informatics Team, Shane A McCarthy, Eerik Aunin, Jim Downie, William Eagles, Noah Gettle, James Gilbert, Ksenia Krasheninnikova, Damon-Lee Pointon, Nathan Riley, Ying Sims, James Torrance, Marcela Uliano-Silva, Chenxi Zhou, Jonathan Wood, Dominic Absolon, Karen Brooks, Joanna Collins, Karen Houliston, Michael Paulini, Sarah Pelan, Thomas Mathers, Camilla Santos, Danil Zilov, Matthieu Muffato, Beth Yates, Tyler Chafin, Cibele Gomes de Sotero Caio, Cibin Sadasivan Baby, Ene Goktan, Paul Davis, Priyanka Surana, Zaynab Butt, Ashish Mittal, Charlie Hathaway, Edward Moulsdale, Kiernan Harding, Logan Howat, Luke Wilson, Ahmad Zuheir bin Zaidon, Caroline Howard, Kerstin Howe, Mark Blaxter, Shane McCarthy, Jonathan M.D. Wood, Fergal Martin, Anna Lazar, Leanne Haggerty, Chiara Bortoluzzi

**Affiliations:** 1 University of Vienna, Centre for Microbiology and Environmental Systems Science, Vienna, 1030, Austria; 2 Leibniz Institute for the Analysis of Biodiversity Change, Museum Koenig Bonn, Bonn, 53113, Germany; 3 Natural History Museum, University of Oslo, Oslo, Norway; 4 Centre for Ecological & Evolutionary Synthesis, University of Oslo, Oslo, Norway; 5 Department of Biological Sciences, University of New Brunswick Saint John, Saint John, Canada; 6 Tjärnö Marine Laboratory, University of Gothenburg, Gothenburg, Sweden; 7 Centre for Coastal Research, University of Agder, Kristiansand, Norway; 8 Tree of Life, Wellcome Sanger Institute, Hinxton, UK; 9 European Molecular Biology Laboratory, European Bioinformatics Institute, Wellcome Genome Campus, Hinxton, UK; 10 SIB Swiss Institute of Bioinformatics, Amphipôle, Quartier UNIL-Sorge, Lausanne, 1015, Switzerland

**Keywords:** Hirudo verbana genome assembly, European Reference Genome Atlas, Biodiversity Genomics Europe, Earth Biogenome Project, leech

## Abstract

*Hirudo verbana* Carena, 1820, commonly known as the southern medicinal leech, is one of several European medicinal leeches, whose full diversity has just recently started to be uncovered. Historically, it has been widely used as a medicinal leech and for centuries it was treated erroneously under the specific name of *Hirudo medicinalis* L. 1758. Recent molecular and taxonomic analyses have revealed subspecific diversity within the morphospecies *H. verbana*. *Hirudo verbana* is a blood-feeding species sucking blood from amphibians, fish, and mammals. It occupies freshwater habitats, typically shallow ponds and lakes. Studies show that this leech species has a "naturally limited microbiome", suggesting it may serve as a powerful model system for the study of gut microbiota. We expect this chromosome-level assembly of *H. verbana* to serve as a high-quality genomic resource for this most famous leech genus and to serve as a foundation to the study of the diversification and biodiversity of European medicinal leeches, as well as their gut-associated symbionts. The genome of *H. verbana* was assembled into two haplotypes through a phased assembly approach; however, only the primary haplotype was designated as the reference genome for annotation and downstream analyses. The entirety of the primary haplotype was assembled into 14 contiguous chromosomal pseudomolecules, including the mitogenome. This chromosome-level assembly encompasses 0.18 Gb, composed of 277 contigs and 27 scaffolds, with contig and scaffold N50 values of 1.3 Mb and 13.4 Mb, respectively.

## Introduction

*Hirudo verbana* Carena, 1820, a member of the *Hirudinidae* family (Annelida: Euhirudinea), inhabits freshwater habitats, most commonly shallow ponds and lakes, where it feeds on amphibians, fish, and mammals ( Elliott & Kutschera, 2011; Vecchioni *et al*., 2025). While it mainly lives underwater, it emerges onto land to lay its spongy cocoons, a reproductive trait shared with other members of the *Hirudinidae* family. *Hirudo* are large cylindrical to dorsoventrally flattened leeches with their whole dorsal surface roughened by small papillae ( Nesemann & Neubert, 1999). Their jaws are monostichodont, with salivary papillae on the jaws being absent. The caudal sucker is medium sized and never exceeds the maximum body diameter. There is no furrow on the upper lip of the cranial sucker. The crop has one pair of short caeca per somite.

*Hirudo verbana* has a characteristic colouration pattern with two broad, reddish, and diffuse paramedian dorsal stripes and a unicoloured greenish to yellow venter with a pair of black marginal stripes ( Utevsky & Trontelj, 2005). In general, the external colouration pattern of European *Hirudo* species is a good discriminatory characteristic ( Trontelj & Utevsky, 2005).

Historically *, H. verbana* (long treated as a colouration type/form of *H. medicinalis* L. 1758) has been, and still is, widely used and trafficked for medicinal and pseudo-medicinal purposes. The species has reliably been found in Switzerland, Austria, Germany, Italy, Slovenia, Croatia, Bosnia and Herzegovina, Serbia, Montenegro, Macedonia, Greece, Hungary, Moldova, Ukraine, Russian Federation, Spain, Turkey, and Uzbekistan ( Arias *et al*., 2021; Utevsky *et al*., 2010) and it was first recognised as separate from *H. medicinalis* by Giacinto Carena ( Carena, 1820) from specimens collected and sent to him from *Lacus Verbanus* (Italian: *Verbano* or *Lago Maggiore*). However, for over a century, his description of *H. verbana* was neglected and it was not until 1999 that Nesemann and Neubert re-established the species’ status ( Nesemann & Neubert, 1999; Trontelj & Utevsky, 2005).

Genetic studies aimed at uncovering the diversity of the European *Hirudo* spp. have revealed that, unlike other European congeners, this species shows significant intraspecific phylogenetic structure ( Trontelj & Utevsky, 2012). Two clades were subsequently recognised: a Western and an Eastern phylogroup. Recently, two further phylogroups have been recognised: an Iberian "typical" and an Iberian "bilineated" ( Arias *et al*., 2021). The latter phylogroup has been described as *Hirudo verbana bilineata* Arias, Surugiu, Carballeira, Popa, Popa & Utevsky, 2021. Thus, it remains likely that unrecognised diversity occurs within the species.

Under laboratory conditions, a leech can feed on two to five times its own mass in one blood meal, which is then digested slowly over many months. Reaching well over 10 cm in length and a mass of several grams, these leeches are an important food source for predators, such as birds and fish. Experiments aimed at discerning differences in biological characteristics of European medicinal leeches have revealed that *H. verbana* has the highest fecundity and juvenile mortality, as well as small juvenile body size when compared to *H. medicialis* and *H. orientalis* ( Petrauskiene *et al*., 2011; Utevsky & Trontelj, 2005) *.*

As other animals, *H. verbana* is host to diverse microbes in its digestive tract. Relevantly, it hosts what has been referred to as a "naturally limited microbiome" consisting of just over a dozen well-defined microbes with a specific localisation. The naturally low diversity microbiome housed by *H. verbana* has led to suggestions of this leech species being a powerful model for the study of microbe-host interactions ( Nelson & Graf, 2012).

As of the 20th of June 2025 *, H. verbana* is not listed on the IUCN Red List of Threatened Species. However, the species is listed on the Appendix II of CITES, establishing quotas for the export of live or frozen wild-taken individuals, on Annex V of the EU Council directive 92/43/EEC on the conservation of natural habitats and of wild fauna and flora, and on several national red lists.

A high-quality reference genome for *H. verbana* will be of great value to different areas of research. First, it will be used for the study of genomic evolution in *Hirudo*, for which other chromosome-level genome references are currently available or in progress ( *H. tianjinensis* Wang, Meng, Jin, Gao, Tong & Liu, 2022, *Hirudo nipponia* Whitman, 1886, and *H. medicinalis*). Second, it will serve as a starting point to understand and unravel cryptic diversity within the morphospecies *H. verbana*. Third, as with previous genomic investigations of *H. medicinalis* ( Kvist *et al*., 2020), this new genome will allow for the exploration of novel putative anticoagulants, which the leech excretes in its saliva to prevent clotting while feeding and which may have medical applications. Finally, as host of digestive-tract symbionts, and as a promising model system for digestive symbioses, a high-quality reference genome for *H. verbana* will contribute to the study of the establishment, maintenance, and crosstalk between the leech host and its symbionts.

The generation of this reference resource was coordinated by the European Reference Genome Atlas (ERGA) initiative’s Biodiversity Genomics Europe (BGE) project, supporting ERGA’s aims of promoting transnational cooperation to promote advances in the application of genomics technologies to protect and restore biodiversity ( Mazzoni *et al*., 2023).

## Materials & methods

ERGA's sequencing strategy includes Oxford Nanopore Technology (ONT) and/or Pacific Biosciences (PacBio) for long-read sequencing, along with Hi-C sequencing for chromosomal architecture, Illumina Paired-End (PE) for polishing (i.e. recommended for ONT-only assemblies), and RNA sequencing for transcriptomic profiling, to facilitate genome assembly and annotation.

### Sample and sampling information

On 11 August 2023, Alejandro Manzano-Marín sampled 9 specimens of *Hirudo verbana* (hermaphrodite monoecious) from an unnamed pond in Vienna by using submerged legs and feet as bait. Species was determined based on the dorsal and ventral colouration patterns following ( Trontelj & Utevsky, 2012). The specimens were identified by Alejandro Manzano-Marín in Vienna, Austria. The specimen selected for sequencing belongs to the so-called Eastern phylogroup and its mitochondrial COI marker is identical to the sequence from a *H. verbana* individual collected from Severynivka in South-western Ukraine (GenBank: JN083798) ( Trontelj & Utevsky, 2012). Sampling permits were not required. Once collected, the specimens were snap frozen and were kept at -80 °C until DNA extraction.

### Vouchering information

For vouchering, adults were relaxed in 10% ethanol and massaged for keeping them stretched. Afterwards, 70% ethanol was increasingly added until fully replaced. For genome and transcriptome sequencing, whole individuals were chopped and snap-frozen in liquid nitrogen. Physical reference materials for the here sequenced specimen have been deposited in the University of Vienna's Zoological collection https://zoologicalcollection.univie.ac.at/ under the accession number UVZC_EV3151.

Tissues (anterior and posterior ends) from the same individual have been deposited in the Biobank of the Leibnitz Institute https://leibniz-lib.de/en/research/research-centres/zmb/bonn-location/biobank.html under voucher IDs ZFMK-TIS-122183 for the anterior end and ZFMK-TIS-122184 for the posterior end.

### Data availability

*Hirudo verbana* and the related genomic study were assigned to Tree of Life ID (ToLID) ‘wcHirVerb1’ and all sample, sequence, and assembly information are available under the umbrella BioProject PRJEB84141. The sample information is available at the following BioSample accession: SAMEA115178104. The genome assembly is accessible from ENA under accession number GCA_965178065.1 and the annotated genome is available through the Ensembl website ( https://projects.ensembl.org/erga-bge/). Sequencing data produced as part of this project are available from ENA at the following accessions: ERX13553724 and ERX13574799. Data used to generate the tables, figures and statistics in this report are available at the following repository: https://doi.org/10.5281/zenodo.17831908. Documentation related to the genome assembly and curation can be found in the ERGA Assembly Report (EAR) document available at https://github.com/ERGA-consortium/EARs/tree/main/Assembly_Reports/Hirudo_verbana/wcHirVerb1. Further details and data about the project are hosted on the ERGA portal at https://portal.erga-biodiversity.eu/data_portal/311461.

### Genetic information

The estimated genome size, based on ancestral taxa, is 0.22 Gb. This is a diploid genome with a haploid number of 13 chromosomes (2n=26). All information for this species was retrieved from Genomes on a Tree ( Challis *et al*., 2023).

### DNA/RNA processing

Protocols for high molecular weight (HMW) DNA extraction developed at the Wellcome Sanger Institute (WSI) Tree of Life Core Laboratory are available on protocols.io ( Denton *et al*., 2023b; Howard *et al.*, 2025). The wcHirVerb1 sample was weighed and triaged ( Jay *et al*., 2023) to determine the appropriate extraction protocol. Tissue from the mid-body was homogenised by powermashing using a PowerMasher II tissue disruptor ( Denton *et al*., 2023a). HMW DNA was extracted using the Automated MagAttract v2 protocol ( Oatley *et al*., 2025a). DNA was sheared into an average fragment size of 12–20 kb following the Megaruptor®3 for LI PacBio protocol ( Bates *et al*., 2023). Sheared DNA was purified by automated SPRI (solid-phase reversible immobilisation) ( Oatley *et al*., 2025b). The concentration of the sheared and purified DNA was assessed using a Nanodrop spectrophotometer and Qubit Fluorometer using the Qubit dsDNA High Sensitivity Assay kit. Fragment size distribution was evaluated by running the sample on the FemtoPulse system.

### Library preparation and sequencing

Library preparation and sequencing were performed at the WSI Scientific Operations core. Libraries were prepared using the SMRTbell Prep Kit 3.0 (Pacific Biosciences, California, USA), according to the manufacturer’s instructions. The kit includes reagents for end repair/A-tailing, adapter ligation, post-ligation SMRTbell bead clean-up, and nuclease treatment. Size selection and clean-up were performed using diluted AMPure PB beads (Pacific Biosciences). DNA concentration was quantified using a Qubit Fluorometer v4.0 (ThermoFisher Scientific) and the Qubit 1X dsDNA HS assay kit. Final library fragment size was assessed with the Agilent Femto Pulse Automated Pulsed Field CE Instrument (Agilent Technologies) using the gDNA 55 kb BAC analysis kit.

The sample was sequenced on a Revio instrument (Pacific Biosciences). The prepared library was normalised to 2 nM, and 15 μL was used for making complexes. Primers were annealed and polymerases bound to generate circularised complexes, following the manufacturer’s instructions. Complexes were purified using 1.2X SMRTbell beads, then diluted to the Revio loading concentration (200–300 pM) and spiked with a Revio sequencing internal control. The sample was sequenced on a Revio 25M SMRT cell. The SMRT Link software (Pacific Biosciences), a web-based workflow manager, was used to configure and monitor the run and to carry out primary and secondary data analysis.

Biotinylated DNA constructs were fragmented using a Covaris E220 sonicator and size selected to 400–600 bp using SPRISelect beads. DNA was enriched with Arima-HiC v2 kit Enrichment beads. End repair, A-tailing, and adapter ligation were carried out with the NEBNext Ultra II DNA Library Prep Kit (New England Biolabs), following a modified protocol where library preparation occurs while DNA remains bound to the Enrichment beads. Library amplification was performed using KAPA HiFi HotStart mix and a custom Unique Dual Index (UDI) barcode set (Integrated DNA Technologies). Depending on sample concentration and biotinylation percentage determined at the crosslinking stage, libraries were amplified with 10–16 PCR cycles. Post-PCR clean-up was performed with SPRISelect beads. Libraries were quantified using the AccuClear Ultra High Sensitivity dsDNA Standards Assay Kit (Biotium) and a FLUOstar Omega plate reader (BMG Labtech). Prior to sequencing, libraries were normalised to 10 ng/μL. Normalised libraries were quantified again and equimolar and/or weighted 2.8 nM pools. Pool concentrations were checked using the Agilent 4200 TapeStation (Agilent) with High Sensitivity D500 reagents before sequencing. Sequencing was performed using paired-end 150 bp reads on the Illumina NovaSeq X. In total, 49x genome coverage in HiFi and 536x genome coverage in HiC data were sequenced to generate the assembly.

### Genome assembly methods

The HiFi reads were assembled using Hifiasm ( Cheng *et al*., 2021) in Hi-C phasing mode, where data were separated into two haplotypes. These haplotypes were then curated to generate a final assembly. The Hi-C reads were aligned to the contigs using bwa-mem2 ( Vasimuddin *et al*., 2019), and contigs were scaffolded with YaHS ( Zhou *et al*., 2023), using the --break option for handling potential misassemblies. The resulting scaffolded assemblies were evaluated using Gfastats ( Formenti *et al*., 2022), BUSCO ( Manni *et al*., 2021), and MERQURY.FK ( Rhie *et al*., 2020).

The mitochondrial genome was assembled using oatk ( Zhou *et al*., 2024) as a single circular contig of 18,667 bp and it is included in the released assembly (GCA_965178065.1). The assembly was decontaminated using the Assembly Screen for Cobionts and Contaminants (ASCC) pipeline (article in preparation). Flat files and maps used in curation were generated in TreeVal ( Pointon *et al*., 2023). Manual curation was primarily conducted using PretextView ( Harry, 2022), with additional insights provided by JBrowse2 ( Diesh *et al*., 2023) and HiGlass ( Kerpedjiev *et al*., 2018). Scaffolds were visually inspected and corrected as described by ( Howe *et al*., 2021). Any identified contamination, missed joins, and mis-joins were corrected, and duplicate sequences were tagged and removed. The curation process is documented at https://gitlab.com/wtsi-grit/rapid-curation (article in preparation). Summary analysis of the released assembly was performed using the ERGA-BGE Genome Report ASM Galaxy workflow ( doi.org/10.48546/workflowhub.workflow.1104.1).

### Cobionts

Bacterial cobionts in the raw data were identified using marker ribosomal RNA loci and then reads corresponding to each identified taxon were extracted and assembled using the MarkerScan pipeline ( Vancaester & Blaxter, 2024).

### Genome annotation methods

A gene set was generated using the Ensembl Gene Annotation system ( Aken *et al*., 2016), primarily by aligning publicly available short-read RNA-seq data from BioSamples SAMN00113400, SAMN15803289, SAMN08595902, SAMN10389976, and SAMN10388009 to the genome. Gaps in the annotation were filled via protein-to-genome alignments of a select set of clade-specific proteins from UniProt ( Consortium, 2019) which had experimental evidence at the protein or transcript level. At each locus, data were aggregated and consolidated, prioritising models derived from RNA-seq data, resulting in a final set of gene models and associated non-redundant transcript sets. To distinguish true isoforms from fragments, the likelihood of each open reading frame (ORF) was evaluated against known metazoan proteins. Low-quality transcript models, such as those showing evidence of fragmented ORFs, were removed. In cases where RNA-seq data were fragmented or absent, homology data were prioritised, favouring longer transcripts with strong intron support from short-read data. The resulting gene models were classified into two categories: protein-coding, and long non-coding. Models that did not overlap protein-coding genes and were constructed from transcriptomic data were considered potential lncRNAs. Potential lncRNAs were further filtered to remove single-exon loci due to their unreliability. Putative miRNAs were predicted by performing a BLAST search of miRBase ( Kozomara *et al*., 2019) against the genome, followed by RNAfold analysis ( Gruber *et al*., 2008). Other small non-coding loci were identified by scanning the genome with Rfam ( Kalvari *et al*., 2018) and passing the results through Infernal ( Nawrocki & Eddy, 2013). Summary analysis of the released annotation was carried out using the ERGA-BGE Genome Report ANNOT Galaxy workflow ( 10.48546/workflowhub.workflow.1096.1).

## Results

### Genome assembly

**Figure 1.**
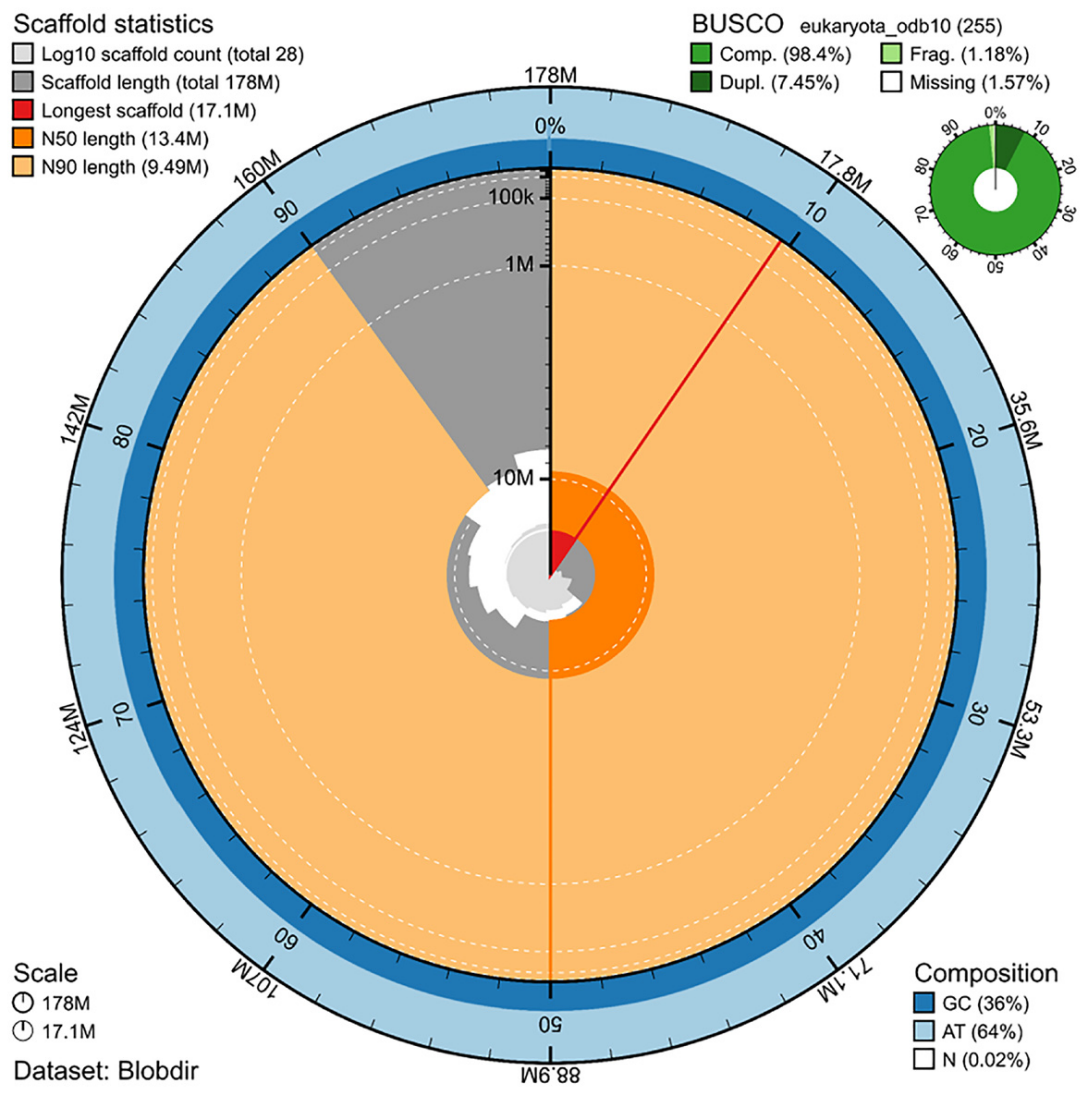
Snail plot summary of assembly statistics. The main plot is divided into 1,000 size-ordered bins around the circumference, with each bin representing 0.1% of the 177,769,257 bp assembly including the mitochondrial genome. The distribution of sequence lengths is shown in dark grey, with the plot radius scaled to the longest sequence present in the assembly (17.1 Mb, shown in red). Orange and pale-orange arcs show the scaffold N50 and N90 sequence lengths (13,422,010 and 9,491,130 bp), respectively. The pale grey spiral shows the cumulative sequence count on a log-scale, with white scale lines showing successive orders of magnitude. The blue and pale-blue area around the outside of the plot shows the distribution of GC, AT, and N percentages in the same bins as the inner plot. A summary of complete, fragmented, duplicated, and missing BUSCO genes found in the assembled genome from the Eukaryota database (odb10) is shown in the top right. The snailplot was generated using the BlobToolKit suite ( Challis *et al*., 2020).

**Figure 2.**
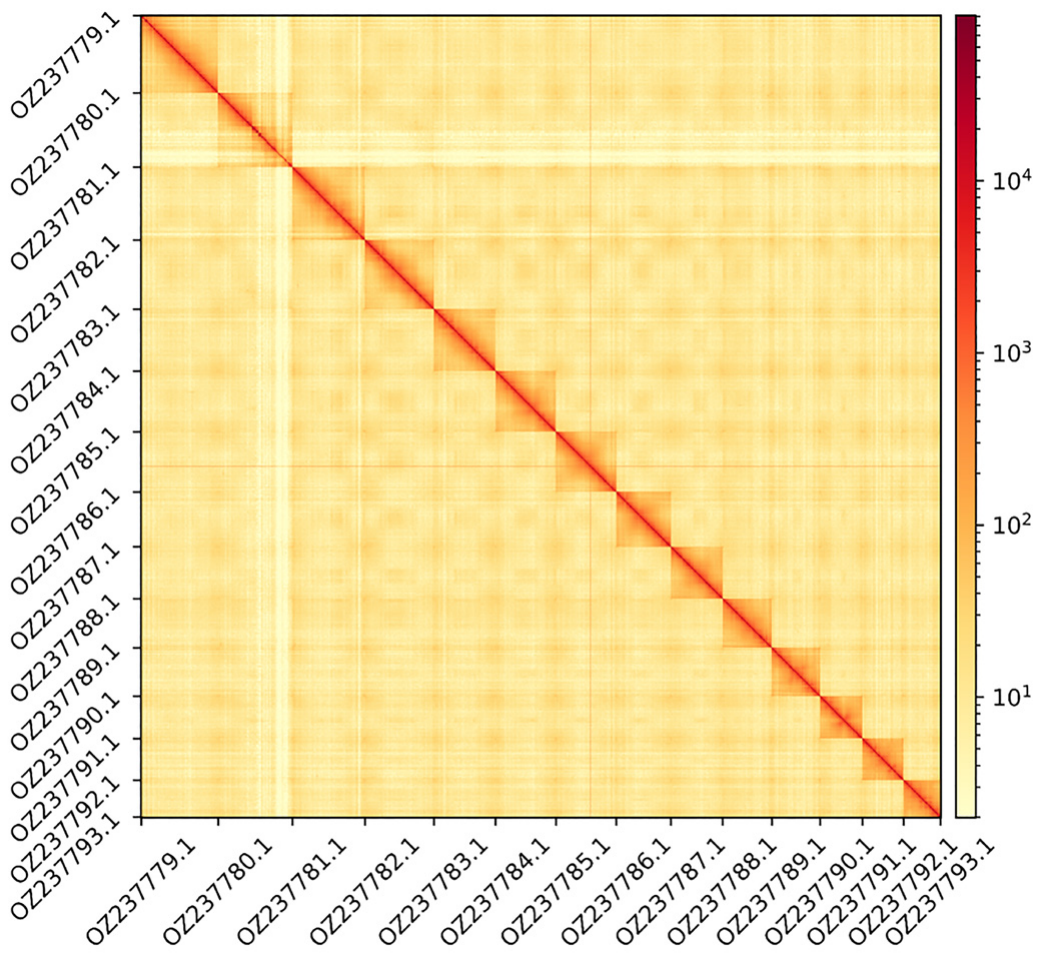
Hi-C contact map showing spatial interactions between regions of the genome. The diagonal corresponds to intra-chromosomal contacts, depicting chromosome boundaries. The frequency of contacts is shown on a logarithmic heatmap scale. Hi-C matrix bins were merged into a 100 kb bin size for plotting. The x-axis and y-axis show the 14 chromosomes and the mitogenome (GenBank: OZ237793.1). The Hi-C contact map was generated using HiCExplorer ( Wolff *et al*., 2020).

The genome assembly has a total length of 177,769,257 bp in 27 scaffolds including the mitogenome ( Figure 1 & Figure 2), with a GC content of 36%. The assembly has a contig N50 of 1,338,446 bp and L50 of 36 and a scaffold N50 of 13,422,010 bp and L50 of 6. The assembly has a total of 250 gaps, totaling 33.5 kb in cumulative size. The single-copy gene content analysis using the Eukaryota database with BUSCO ( Manni *et al*., 2021) resulted in 98.4% completeness (91.0% single and 7.5% duplicated). 77.1% of reads k-mers were present in the assembly and the assembly has a base accuracy Quality Value (QV) of 55.1 as calculated by Merqury ( Rhie *et al*., 2020).

### Genome annotation

The genome annotation consists of 15,195 protein-coding genes with associated 23,608 transcripts, in addition to 8,272 non-coding genes ( Table 1). Using the longest isoform per transcript, the single-copy gene content analysis using the Eukaryota database with BUSCO resulted in 97.6% completeness. Using the Metazoa-v2.0.0.h5 database for OMArk ( Nevers *et al*., 2025) resulted in 93.3% completeness and 64.1% consistency ( Table 2).

**Table 1.** Statistics from assembled gene models.

	No. genes	No. transcripts	Mean gene length (bp)	No. single-exon genes	Mean exons per transcript
**mRNA**	15,195	23,608	6,092	274	8.4
**pseudogene**	0.0	0.0	0.0	0.0	0.0
**snoRNA**	193	193	179	193	1.0
**lncRNA**	1,536	1,704	2,583	5	2.5
**tRNA**	5,709	5,709	80	5,709	1.0
**snRNA**	678	678	155	678	1.0
**rRNA**	155	155	226	155	1.0
**scRNA**	1	1	134	1	1.0
**Other ncRNA**	6,896	32,203	79 – 152	6,896	1.0 – 7.8

**Table 2.** Annotation completeness and consistency scores calculated by BUSCO run in protein mode (eukaryota_odb10) and OMArk (Metazoa-v2.0.0.h5).

	Complete	Singular	Duplicated	Fragmented	Missing
**BUSCO**	249 (97.6%)	0 (0.0%)	249 (97.6%)	3 (1.2%)	3 (1.2%)
**OMArk**	2,010 (93.3%)	352 (16.3%)	1,658 (77.0%)	-	143 (6.7%)
	Consistent	Inconsistent	Contaminants	Unknown	
**OMArk**	19,492 (64.1%)	3,024 (10.0%)	0 (0.00%)	7,874 (25.9%)	

### Cobionts

Within the raw data for *Hirudo verbana* we identified sequences derived from seven distinct bacterial families. Because we do not, *a priori*, know the relationships between these bacteria and the leech, we use the term “cobionts”: they could be mutualist symbionts, members of the microbiome, pathogens or chance associations with environmental organisms. After sorting prokaryote-derived reads into bins (containing from 0.2% to 8% of raw reads) with MarkerScan ( Vancaester & Blaxter, 2024) we assembled genomes from these bacterial families ( Table 3). Three of the bins had assembled spans (>10 Mb) that suggested that more than one strain or species had been co-assembled; these bins had high BUSCO duplication scores.

**Table 3.** Cobionts. Information is taken directly from MarkerScan at https://tolqc.cog.sanger.ac.uk/erga-bge/annelids/Hirudo_verbana.

specimen	family	original classified reads			original assembly				re-assembly			
		count	(%)	BUSCO	BUSCO	contigs	contig length	number of reads	BUSCO	contigs	contig length	number of reads
wcHirVerb1	Chitinophagaceae	4,553	0.38	C:99.2%[S:1.6%,D:97.6%],F:0.0%,M:0.8%,n:124	C:98.3%[S:92.7%,D:5.6%],F:0.8%,M:0.9%,n:124	3	3.33Mb	2,843	C:99.2%[S:62.1%,D:37.1%],F:0.0%,M:0.8%,n:124	24	4.53Mb	5,130
wcHirVerb1	Comamonadaceae	50,786	4.21	C:91.9%[S:0.9%,D:91.0%],F:1.6%,M:6.5%,n:688	C:32.1%[S:19.0%,D:13.1%],F:0.9%,M:67.0%,n:688	42	1.65Mb	4,219	C:91.8%[S:3.1%,D:88.7%],F:1.2%,M:7.0%,n:688	64	26.16Mb	116,895
wcHirVerb1	Myxococcaceae	3,696	0.31	C:93.5%[S:4.8%,D:88.7%],F:5.6%,M:0.9%,n:124	C:18.5%[S:15.3%,D:3.2%],F:3.2%,M:78.3%,n:124	14	0.99Mb	283	C:91.1%[S:41.9%,D:49.2%],F:7.3%,M:1.6%,n:124	6	4.99Mb	9,991
wcHirVerb1	Phyllobacteriaceae	11,363	0.94	C:100.0%[S:1.2%,D:98.8%],F:0.0%,M:0.0%,n:432	C:99.1%[S:22.5%,D:76.6%],F:0.2%,M:0.7%,n:432	50	6.96Mb	6,512	C:99.7%[S:23.1%,D:76.6%],F:0.0%,M:0.3%,n:432	72	12.59Mb	20,807
wcHirVerb1	Pseudobdellovibrionaceae	473	0.04	C:94.3%[S:0.8%,D:93.5%],F:2.4%,M:3.3%,n:124	C:0.8%[S:0.8%,D:0.0%],F:0.0%,M:99.2%,n:124	1	0.04Mb	6	C:92.7%[S:87.1%,D:5.6%],F:3.2%,M:4.1%,n:124	4	2.44Mb	5,111
wcHirVerb1	Rikenellaceae	1,152	0.1	C:94.8%[S:0.2%,D:94.6%],F:0.9%,M:4.3%,n:541	C:5.7%[S:5.7%,D:0.0%],F:0.4%,M:93.9%,n:541	3	0.18Mb	40	C:94.9%[S:47.9%,D:47.0%],F:0.7%,M:4.4%,n:541	13	5.03Mb	5,804
wcHirVerb1	Sphingobacteriaceae	15,660	1.3	C:92.4%[S:0.1%,D:92.3%],F:0.5%,M:7.1%,n:1068	C:87.9%[S:0.2%,D:87.7%],F:0.3%,M:11.8%,n:1068	42	11.49Mb	12,985	C:92.3%[S:4.1%,D:88.2%],F:0.5%,M:7.2%,n:1068	11	17.41Mb	30,309

## Data Availability

The underlying data has been deposited in the European Nucleotide Archive (ENA), accession number PRJEB84141: https://www.ebi.ac.uk/ena/browser/view/PRJEB84141, and Ensembl, accession number GCA_965178065.1: https://ftp.ebi.ac.uk/pub/ensemblorganisms/Hirudo_verbana/GCA_965178065.1/. Data used to generate the tables, figures and statistics in this report are available at the following repository: https://doi.org/10.5281/zenodo.17831908 ( Manzano-Marín *et al.*, 2025). All data are available under the terms of the Creative Commons Zero “No rights reserved” data waiver (CC0 1.0 Public domain dedication).
