# Supplementary material for: ERGA-BGE reference genome of Hirudo verbana, a once neglected freshwater haematophagous European medicinal leech
Source: Open Res Eur. 2026 Feb 10;5:395. Originally published 2025 Dec 22. [Version 2] doi: 10.12688/openreseurope.21672.2 (PMC12820476; doi:10.12688/openreseurope.21672.2)
Supplement: Peer review history

# Reviewer report: Reviewer response for version 2

**DOI:** 10.21956/openreseurope.24798.r69390

**Published:** 2026-02-11

**Authors:** Christian Mueller

**Affiliations:** 1 University of Greifswald, Greifswald, Germany

I do not have any additional comments.

Are sufficient details of methods and materials provided to allow replication by others?

Yes

Is the rationale for creating the dataset(s) clearly described?

Yes

Are the datasets clearly presented in a useable and accessible format?

Yes

Are the protocols appropriate and is the work technically sound?

Yes

Reviewer Expertise:

animal physiology; molecular biology; leech biology and physiology; anticoagulants

I confirm that I have read this submission and believe that I have an appropriate level of expertise to confirm that it is of an acceptable scientific standard.

# Reviewer report: Reviewer response for version 1

**DOI:** 10.21956/openreseurope.23444.r67473

**Published:** 2026-01-20

**Authors:** Christian Mueller

**Affiliations:** 1 University of Greifswald, Greifswald, Germany

**Short Summary:**

 The author(s) submitted a manuscript that describes the generation, assembly and quality analysis of the almost complete chromosomal genome and the complete mitochondrial genome of *Hirudo verbana*, one of the European medicinal leech species. In addition the genomes of a total of seven different bacterial cobionts of *Hirudo verbana* were determined as well. 

**General Impression**

 The manuscript is part of the European Reference Genome Atlas (ERGA) initiative in general and the  Biodiversity Genomics Europe (BGE) project in particular. It addresses a topic that is of great interest for biologist and ecologists on one hand and for physicians and non-medical practitioners on the other hand, but beyond that also for a broad audience as it helps to shed light on the diversity of life. The manuscript is in line with an increasing number of publications on complete genomes of different hematophagous and non-hematophagous leech species.  

 I fully appreciate the efforts of the authors, and I have no doubts that all experiments and analyses were performed with accuracy. The manuscript is well written, the results and data are clearly presented. In that context I strongly recommend its publication. There are only a few minor remarks that need to be addressed.

**Minor remarks:**

 1) The statement on the distribution of *H. verbana* across Europe dates back to Utevsky et al. 2010, but in the meantime the species was reliably described also in Slovakia (Cséfalvay et al. 2017; Folia faunistica Slovaca 22:63-66) and in Romania (Popa et al. 2024; Diversity 16, 726.

 https://doi.org/10.3390/d16120726). Please include the new references.

 On the other hand, the "reliable" descriptions of *H. verbana* in Germany are very likely artifacts and the result of accidental or intended releases of leeches to the wild, but the species does not belong to the natural fauna of Germany. The presence of stable reproductive populations of *H. verbana* in Germany is hence highly questionable.

 2) Draft genomes of *H. medicinalis* were published in 2020 both by Kvist et al. and by Babenko et al. (Babenko et al. 2020; BMC Genomics 21:331; https://doi.org/10.1186/s12864-020-6748-0). Please acknowledge and cite also the contribution of the Russian colleagues. In addition, a draft genome of *H. verbana* was generated and a respective publication deposited in BioRxiv (Paulsen et al. 2020, https://doi.org/10.1101/2020.12.08.416024), but for unknown reasons never officially published. However, I recommend to also cite the contribution of the US colleagues.

 3) I recommend to include a photograph of *H. verbana*, just as an eye-catcher.

 4) The manuscript lacks any further analyses like the identification of putative anticoagulants. At least a short comparison with the genomes of Asian medicinal leeches would, in my opinion, significantly  enhance the strength and the attractiveness of the manuscript. Please consider.

Are sufficient details of methods and materials provided to allow replication by others?

Yes

Is the rationale for creating the dataset(s) clearly described?

Yes

Are the datasets clearly presented in a useable and accessible format?

Yes

Are the protocols appropriate and is the work technically sound?

Yes

Reviewer Expertise:

animal physiology; molecular biology; leech biology and physiology; anticoagulants

I confirm that I have read this submission and believe that I have an appropriate level of expertise to confirm that it is of an acceptable scientific standard.

# Response

**Published:** 2026-01-20

**Authors:** Chiara Bortoluzzi

**Affiliations:** Swiss Institute of Bioinformatics, Lausanne, Vaud, Switzerland

We thank the reviewer for their positive feedback and comments. All comments are addressed in the revised manuscript as follow:   

 1.  You are absolutely right. Slovakia and Romania have been added to the list of countries and references are now added in the main text.  “The species has reliably been found in Switzerland, Austria,Italy, Slovenia, Croatia, Bosnia and Herzegovina, Serbia, Montenegro, Macedonia, Greece, Hungary, Moldova, Ukraine, Romania, Russian Federation, Slovakia, Spain, Turkey, and Uzbekistan ( Arias et al., 2021; Utevsky et al., 2010; Cséfalvay et al. 2017; Popa et al. 2024)” On the other hand, the "reliable" descriptions of H. verbana in Germany are very likely artifacts and the result of accidental or intended releases of leeches to the wild, but the species does not belong to the natural fauna of Germany. The presence of stable reproductive populations of H. verbana in Germany is hence highly questionable. With the term ‘reliable’, we wanted to convey rather the message of bona fide descriptions of H. verbana (taxonomically speaking), and did not intend to imply that there are in fact “reproductive populations” of H. verbana there. For example, even in Austria, H. medicinalis is what one would expect, but the sequenced population can reliably be traced to an artificial historical introduction that has developed into a very isolated stable reproductive population. We have however decided to remove Germany from the list, as we recognise the statement could in fact be read as such.

 2. You are right. In its original text, we thought more of the highlight of the use of these genomes to identify putative novel anticoagulants (the focus of Kvist et. al 2020). Babenko et al. 2020 rather focused on draft transcriptomes to extract this information. This is why we originally omitted the citation. We have nevertheless added it now. We are aware of the previous draft genome pre-print of a specimen identified as H. verbana. We did not include this citation as we do not delve into previous genomic investigations of H. verbana. We have thus added a small mention to it.

 3. We added a photograph of H. verbana.

 4. We did not perform any further analyses on the identification of putative anticoagulants, as this paper is thought of as a genome report to make the reference genome and associated resources avaiable to the broader community. Thus, such analysis would go beyond the scope of this report. Other researchers interested in this area are welcome to use this genomic resource for this purpose.

# Reviewer report: Reviewer response for version 1

**DOI:** 10.21956/openreseurope.23444.r66970

**Published:** 2026-01-12

**Authors:** Naim Saglam

**Affiliations:** 1 Firat University, Elazığ, Turkey

This study follows the highest standards of modern genomics. The presentation of a "reference genome" for a species ( *Hirudo verbana*) with taxonomic complexity and medicinal importance enhances the value of the article. However, it is important to consider the following points:

 1. While the genome is stated as 0.18 Gb in the abstract, the estimated size is given as 0.22 Gb in the "Genetic Information" section. A brief explanation of whether this difference is due to repetitive sequences or regions lost during compilation would be helpful.

 2. The study was conducted on samples collected from an unnamed pond in Vienna. Providing GPS coordinates for the "unnamed pond" in Vienna, if possible, would be beneficial for reproducibility.

 3. *Hirudo* species are generally included in CITES Appendix II. Clarifying the exemption status regarding the collection of this species in Austria and providing an explanation of whether the necessary permits for this research were obtained would be helpful.

 4. The inclusion of the genomes of seven different bacterial families, along with the leech genome, in this article enhances the biological value of the study. However, a brief discussion of the relationships between the bacterial families in Table 3 and other bacteria found in the leech's known gut microbiota would make the text more informative. 

 5. The article appears to lack a discussion section. Considering previous molecular studies conducted on *Hirudo* species, a discussion section should be included to enhance the article's clarity and informativeness.

Are sufficient details of methods and materials provided to allow replication by others?

Yes

Is the rationale for creating the dataset(s) clearly described?

Yes

Are the datasets clearly presented in a useable and accessible format?

Yes

Are the protocols appropriate and is the work technically sound?

Yes

Reviewer Expertise:

Leeches, Molecular evolution, RN/DNA, Transcriptomics and Fish diseases.

I confirm that I have read this submission and believe that I have an appropriate level of expertise to confirm that it is of an acceptable scientific standard.

# Response

**Published:** 2026-01-20

**Authors:** Chiara Bortoluzzi

**Affiliations:** Swiss Institute of Bioinformatics, Lausanne, Vaud, Switzerland

We thank the reviewer for their positive feedback and additional comments. All comments have been addressed in the new revised manuscript as follow: 

 1. The difference in genome size between what reported in the abstract and what reported in the ‘Genetic information’ section is due to the fact the while the first value (0.18) is the actual size of the sequenced genome, the second value (0.22) is an estimate obtained from ancestral taxa. This estimate is used prior to sequencing to help calculate sequencing effort (e.g. library prep reagents and number of Revio flow cells) and it is known that the value might not be exact, but it is an educated guess. The assumption that a species might have its genome size within the range described for its taxonomic group is the basis for GoaT estimates: the median of genome sizes are computed from taxa with direct measurements and propagated to the closest common ancestor, then back to fill the missing data. More information on how GoaT calculates ancestral values to propagate can be found in https://wellcomeopenresearch.org/articles/8-24/v1. K-mer genome sizes are part of our pipelines and are used routinely to assess the need for top-up sequencing during the process of producing a genome. The final assessment of a sequence-based genome size is its assembly span, which, together with K-mer inferred genome size measurements, are not the definitive amount of DNA in cells, but a reference to the size of chromosome assemblies. We reformulated this part to make it more clear. 

 2. We now provide the general GPS coordinates in the main text. However, the exact GPS coordinates were omitted in order to protect the collection site from exploitation, as this is a problem when reporting collection sites for European medicinal leeches.

 3. There are currently only two Hirudo species currently listed in CITES Appendix II: Hirudo medicinalis, and Hirudo verbana. Sometimes Hirudo spp. are treated as a whole by CITES agents as they cannot generally assess the specific species affiliation. The appendix II, as it relates to these two species, does not relate to the collection of the species in the wild, but rather it controls its trade, “Appendix II lists species that are not necessarily now threatened with extinction but that may become so unless trade is closely controlled. It also includes so-called "look-alike species", i.e. species whose specimens in trade look like those of species listed for conservation reasons (see Article II, paragraph 2 of the Convention). International trade in specimens of Appendix-II species may be authorized by the granting of an export permit or re-export certificate. No import permit is necessary for these species under CITES (although a permit is needed in some countries that have taken stricter measures than CITES requires). Permits or certificates should only be granted if the relevant authorities are satisfied that certain conditions are met, above all that trade will not be detrimental to the survival of the species in the wild. (See Article IV of the Convention)” ( https://cites.org/eng/app/index.php). And “2. Appendix II shall include: (a) all species which although not necessarily now threatened with extinction may become so unless trade in specimens of such species is subject to strict regulation in order to avoid utilization incompatible with their survival; and (b) other species which must be subject to regulation in order that trade in specimens of certain species referred to in sub-paragraph (a) of this paragraph may be brought under effective control. ” ( https://cites.org/eng/disc/text.php#II). Therefore, its collection is not controlled, but rather its trade, establishing quotas per country ( https://www.speciesplus.net/species#/taxon_concepts/6167/legal). See also Commission Reg. (EU) 2023/966 of 15 May 2023 ( https://eur-lex.europa.eu/legal-content/EN/TXT/PDF/?uri=CELEX:32023R0966).  In fact, Hirudo verbana is included as it is considered a "look-alike species" to its congener H. medicinalis. In Austria, no specific regulations control its collection, as this was also not done in any recognised natural reserve and species is not recognised as one protected from its collection.  Thus, permits were only required for its shipping from Austria and into the UK. These CITES permits were granted with numbers AT-23–0027 (Austria, export) and 24GBIMPD10R7G (UK, import).  We added a few sentences about this in the main text in the section ‘Sample and sampling information’.

 4. the bacterial MAGs reported were done so in the interest of reporting this finding, and given previous reports of the presence of some of these taxa as members of the microbiome of H. verbana. However, these have not been further analysed, as they were not the focus of this genome data report and are being currently fully analysed for a separate work focusing on the genomics, diversity, metabolic potential, and evolution of these entities.

 5. We are aware that the article lacks a discussion section, but this is due to the fact that this is a Data Note, whose scope is to present data, in our case the genome assembly of Hirudo verbena. Unfortunately, we cannot add such a section due to a constraint in the article format.
